# Supplementary material for: A Critical View of the Application of the APEX Software (Aqueous Photochemistry of Environmentally-Occurring Xenobiotics) to Predict Photoreaction Kinetics in Surface Freshwaters
Source: Molecules. 2019 Dec 18;25(1):9. doi: 10.3390/molecules25010009 (PMC7017383; doi:10.3390/molecules25010009)
Supplement: Supplementary file 1 [file molecules-25-00009-s001.zip › 3.2.4_gcc-4.4.0.zip!/3.2.4_gcc-4.4.0/3.2.4_gcc-4.4.0/doc/octave/html/interpreter/Line-Styles.html]

Line Styles - Untitled


Next: Marker Styles,
Previous: Colors,
Up: Advanced Plotting


---

#### 15.2.5 Line Styles

Line styles are specified by the following properties:

`linestyle`: May be one of `"-"`: Solid lines. `"--"`: Dashed lines. `":"`: Points. `"-."`: A dash-dot line. `linewidth`: A number specifying the width of the line. The default is 1. A value of 2 is twice as wide as the default, etc.
